# UHRF1-induced connexin26 methylation is involved in hearing damage triggered by intermittent hypoxia in neonatal rats

**DOI:** 10.1515/med-2023-0650

**Published:** 2023-02-25

**Authors:** Xingang Zhang, Jishan Zheng, Huiqing Xu, Zhaoxin Ma

**Affiliations:** Department of Otorhinolaryngology-Head and Neck Surgery, Shanghai East Hospital, Tongji University School of Medicine, Pudong New Area, Shanghai, 200120, China; Department of Otorhinolaryngology-Head and Neck Surgery, Ningbo Women and Children’s Hospital, Ningbo, Zhejiang Province, 315012, China; Department of Pediatrics, Ningbo Women and Children’s Hospital, Ningbo, Zhejiang Province, 315012, China; Department of Otorhinolaryngology-Head and Neck Surgery, Tongji University School of Medicine, Pudong New Area, No. 150 Jimo Road, Shanghai, 200120, China

**Keywords:** hearing loss, intermittent hypoxia, ubiquitin-like with plant homeodomain (PHD) and ring finger domains 1, Connexin26, methylation

## Abstract

Ubiquitin-like with plant homeodomain and ring finger domains 1 (UHRF1) promotes the maintenance of established patterns of DNA methylation in mammalian cells. Extensive methylation of connexin26 (COX26) during hearing impairment has been demonstrated. The present study aims to determine whether UHRF1 can induce the methylation of COX26 in cochlea damaged by intermittent hypoxia (IH). After the establishment of the cochlear injury model through IH treatment or isolation of the cochlea containing Corti’s organ, pathological changes were observed via HE staining. Expressions of COX26 and UHRF1 were detected by quantitative reverse-transcription polymerase chain reaction and Western blot. The effect of COX26 methylation levels was analyzed by methylation-specific PCR (MSP). Phalloidin/immunofluorescence staining was used to observe structural changes. The binding relationship between UHRF1 and COX26 was verified by chromatin immunoprecipitation. IH caused cochlear damage, accompanied by increased methylation of COX26 and expression of UHRF1 in the cochlea of neonatal rats. CoCl_2_ treatment caused the loss of cochlear hair cells, downregulation and hypermethylation of COX26, abnormal upregulation of UHRF1, and disordered expressions of apoptosis-related proteins. UHRF1 in cochlear hair cells binds to COX26, and its knockdown upregulated COX26 level. Overexpressed COX26 partially alleviated the CoCl_2_-caused cell damage. UHRF1 induces COX26 methylation and aggravates the cochlear damage caused by IH.

## 1 Introduction

Hearing impairment is a relatively common type of neonatal disability. With the improvement of obstetric medical treatment and the popularization of hearing screening, the incidence of neonatal hearing impairment has been effectively reduced [1]. However, according to the statistics, 1–3.47 infants will have hearing impairment in every 1,000 live births [2], and the incidence is higher in countries with slow economic development. Due to a lack of external sound stimulation, infants with hearing impairments fail to enter the language learning stage normally after birth, which seriously affects the physical and mental health of infants and young children. Among the pathogenic factors identified, intermittent hypoxia (IH) is the most common risk factor for hearing impairment [3]. Newborns, especially premature babies, have poorly developed respiratory systems and are prone to hypoxia. Sufficient oxygenation is essential for normal cochlear function, in contrast to severe hypoxia, which can cause irreversible damage to the outer hair cells and stria vascularis of the cochlea [3,4]. Therefore, how to intervene early and reduce the risk of IH in time is the focus of the current research.

With the in-depth exploration of pathogenesis, screening specific molecular targets has become a key research direction to improve disease diagnosis and guide disease treatment [5]. In the past decade, molecular diagnostic technology has become the most important screening method to assess hearing impairment [6,7]. Ubiquitin-like with plant homeodomain and ring finger domains 1 (UHRF1) is a vital epigenetic regulator, whose expression is evidently increased in many tumors, including breast cancer, bladder cancer, and liver cancer [8]. In addition, UHRF1 plays a critical role in ensuring the correct replication of DNA methylation and regulating the function of heterochromatin and gene expression. The specific regulation of UHRF1 is realized through transferring chemotactic DNA methyltransferase 1 (DNMT1) to the bifurcation of DNA replication, thus maintaining DNA methylation [9,10]. Smets et al. found that patients with hereditary sensory and autonomic neuropathies accompanied by hearing impairment have DNMT1 mutations, which damage UHRF1 interaction and lead to undermethylation [11]. In view of this, whether the changes in UHRF1 expression affect the progress of hearing impairment still needs to be further expounded.

GJB2 gene mutation is one of the most common clinical causes of hereditary nonsyndromic deafness [12]. Connexin26 (COX26) is the most pivotal gap junction protein encoded by the GJB2 gene and is highly expressed in the cochlear gap junction (Gap Junction) [13]. The defect of COX26 not only results in congenital deafness but also can lead to delayed deafness [14]. It has been documented that COX26 is abnormally methylated in the course of hearing impairment [15,16]. However, the molecular mechanism of this process needs further analysis and exploration.

Based on the above literature analyses, this study is committed to unveiling the effect of UHRF1 on the process of hearing damage induced by IH, and clarifying whether the effect of UHRF1 is related to the regulation of COX26 methylation.

## 2 Methods

### 2.1 Establishment of IH neonatal rat model

The 15-day-pregnant Sprague-Dawley (SD) rats (*n* = 4) were purchased from Beijing Vital River Laboratory Animal Technology Co., Ltd. Two hours (h) after the rats gave birth, all newborn rats were transferred together, with ten pups randomly divided into the control group (*n* = 5) and the IH group (*n* = 5), and the remaining pups applied for follow-up *in vitro* studies (*n* = 18). With reference to Alex’s treatment method [17], pups in the IH group were subjected to hypoxia treatment from 0 to 14 days after birth in a specially designed oxygen chamber. In brief, the pups were first exposed to 50% O_2_ for 30 min (min), followed by three hypoxia–reoxygenation treatments: the O_2_ concentration was reduced to 12% (lasted 1 min) and restored to 50% (lasted 10 min). The pups in the IH group received the above treatment once a day. For the rest of the time, the pups in the IH group and the control group were kept in a regular indoor air environment. Auditory brainstem response (ABR) thresholds were determined just before euthanization [18]. On the 56th day of birth, the SD rats in both groups were anesthetized with 4% isoflurane gas (R510-22; RWD, China). The intact cochlear tissue of the rats was taken out.

### 2.2 Cochlea collection and cochlear organotypic culture

The pups used in the *in vitro* study were also anesthetized with 4% isoflurane on the fourth day after birth. According to Wang’s instruction [19], the skull of SD pups was opened, and the temporal bone tissue was extracted. Excess tissues and bones were removed. The spiral ligament and the surrounding internal nerve fibers along the loop of the worm axis were separated to cut out the entire cochlea containing Corti’s organ. The basement membrane of the cochlea was placed in a petri dish coated with polylysine. Dulbecco’s modified eagle medium (DMEM)/F12 (D6421; Sigma–Aldrich, USA) containing 10% fetal bovine serum (fetal bovine serum (FBS); 12007C) was added to provide the material basis for the normal growth of the cochlea. Slow and careful operation helped to maintain the natural curvature of the cochlea. The cochlea was randomly divided into three groups: the control group, the CoCl_2_-400 group, and the CoCl_2_-400 + 5-Aza-CdR group. The cochlea in the control group was only subjected to normal culture. After 24 h of routine culture, the cochlea in the CoCl_2_-400 group received 400 μM CoCl_2_ (221015; Sigma–Aldrich) for 48 h of intervention [19]. The cochlea in the CoCl_2_-400 + 5-Aza-CdR group was processed with CoCl_2_ and then further treated with 50 µM 5-Aza-CdR (189825; Sigma–Aldrich) for 48 h [20]. The above animal treatment was approved by the Institutional Animal Care and Use Committee (ZJCLA-IACUC-20040110).

### 2.3 Cochlear hair cells

The basic medium for cochlear hair cells HEI-OC1 (ml-A355; MlBio, China) was DMEM (56499C; SAFC, USA), with 10% FBS added to the complete medium. HEI-OC1 cells received the following seven different treatments: normal culture in the control group; treatment with 400 μM CoCl_2_ for 48 h in CoCl_2_ group; transfection with negative control of short hairpin RNA (sh-NC) for 48 h followed by treatment with 400 μM CoCl_2_ for 48 h in CoCl_2_ + sh-NC group; transfection with sh-UHRF1 plasmid for 48 h followed by treatment with 400 μM CoCl_2_ for 48 h in the CoCl_2_ + sh-UHRF1 group; treatment with CoCl_2_ and then with 50 µM 5-Aza-CdR reagent for 48 h in the CoCl2 + 5-Aza-CdR group; transfection with negative control (NC) for 48 h, followed by 400 μM CoCl_2_ intervention for 48 h in the CoCl_2_ + NC group; and transfection with COX26 overexpression plasmid for 48 h followed by 400 μM CoCl_2_ intervention for 48 h in the CoCl_2_ + COX26 group. At 0, 12, and 24 h after the treatments, the confluency of HEI-OC1 cells was observed under a microscope (200×, LSM900; ZEISS, Germany).

The COX26 overexpression plasmid, NC (blank vector), sh-UHRF1 (silenced sequence ACGTCATTTACCACGTGAAATAC), and sh-NC (blank shRNA) were synthesized by GenePharma. After reactions with Roche X-tremeGENE™ 360 Transfection Reagent (XTG360-RO), HEI-OC1 cells were transfected with the aforementioned plasmids, respectively. The specific transfection efficiency was tested 48 h post-transfection by quantitative reverse-transcription polymerase chain reaction (qRT-PCR) and Western blot experiments.

### 2.4 Hematoxylin–eosin (HE) staining

In animal experiments, the cochlea tissues of SD rats in the control and IH groups were stripped and immediately immersed in paraformaldehyde (4%) for 24 h. Ethylenediaminetetraacetic acid decalcification solution (E1171; Solarbio, China) was applied to effectively elute calcium in the cochlear tissue. The cochlear tissue was embedded in an optimal cutting temperature compound (4583; SAKURA, USA) and cut into tissue sections. Cochlear sections were stained by HE from the Solarbio HE kit (G1120). The mounted sections were enlarged between 40 and 100 times under a microscope to observe the pathological changes of the cochlea.

### 2.5 Phalloidin staining

In order to observe the structure of Corti’s organ and the apoptosis of hair cells more clearly, Phalloidin staining method was employed [3]. Briefly, the cochlea extracted from the *in vitro* experiment was fixed with paraformaldehyde for 20 min. The cochlea sections were stained with the red tetramethylrhodamine labeled Phalloidin (AAT-23102; AAT Bioquest, USA) for 1 h, followed by slow washing. The staining solution was replaced with a 4,6-diamidino-2-phenylindole dihydrochloride (DAPI) reagent to stain the nucleus. After the slide with the fluorescent mounting solution was mounted, the staining results were observed under a microscope (400×), and the number of apoptotic hair cells was calculated.

### 2.6 Immunofluorescence staining

The cochlea harvested from the *in vitro* experiment was sliced according to the method mentioned in HE staining. 0.1% Triton X-100 (×100; Sigma–Aldrich) was dropped on the fixed sections. Following 15 min of permeabilization, 5% normal goat serum (SL038; Solarbio, China) was used to seal the tissue sections. After 1 h, the anti-COX26 antibody (ab59020; Abcam, UK) diluted 200 times and covered with the cochlea sections overnight (4°C). After the surface liquid was sucked, the cochlea sections were further reacted with the labeled Donkey Anti-Goat antibody (fluorescently labeled Alexa Fluor^®^ 647; dilution ratio: 1/500; ab150135; Abcam) for 1 h, followed by being stained with DAPI staining and mounted with fluorescent mounting fluid. The stained sections were observed under a microscope at a magnification of ×400, and the expression of COX26 in the Corti’s organ was detected.

### 2.7 qRT-PCR

To test the messenger RNA (mRNA) levels of COX26 and UHRF1 in cochlear tissue/hair cells, a total RNA extraction reagent (T9424; Sigma–Aldrich) was added to the tissue homogenate/cell suspension. Next, the total RNAs were collected, and the concentration of which was determined. Then, the RNAs were converted into complementary DNA (cDNA) using a BIO-RAD iScript cDNA synthesis kit (170-8890) and T100-Thermal Cycler, followed by qRT-PCR. With reference to a previous report [20], the convenient and accurate SYBR Green fluorescent dye detection method (QR0100; Sigma–Aldrich) was adopted. COX26, UHRF1, and specific primers for reference genes (Table 1; Sangon Biotech), as well as cDNA, were mixed with the fluorescent dyes in the kit and then added to the ABI StepOne fluorescent qRT-PCR machine. The relative mRNA levels were finally calculated using 2^−ΔΔCT^ method.

**Table 1:** Primers for qRT-PCR

**Gene**	**Forward primer (5**′**–3**′)	**Reverse primer (5**′**-3**′)
rno-Connexin26	TTGAAACCACTCAGAGGGCA	GCTCTGTAGTGTGCCCCAAT
mmu-Connexin26	CATTTCGGACCAACCCAGGA	TGCCCCAATCCATCTTGTCC
rno-UHRF1	CCGGTATTGCTACGGGGTTT	CACATGATGCCGATGTGCTG
mmu-UHEF1	GGTGGGGTTCAGTACATCGG	GCTCTGGATAGTTGACATGGT
GAPDH	TGTGGGCATCAATGGATTTGG	ACACCATGTATTCCGGGTCAAT

### 2.8 Western blot

Western blot was another basic molecular biology experimental method in this study. Similar to the prior literature report [21], the total proteins in the cochlear tissue/hair cells were decomposed and extracted using radio immunoprecipitation assay Lysis Buffer (P0013C; Beyotime, China). BCA Protein Assay kit (P0010; Beyotime) and Thermo Scientific Multiskan SkyHigh were applied for the determination of protein concentration. After being separated by the sodium dodecyl sulfate-polyacrylamide gel electrophoresis gel, the proteins were transferred into the Roche polyvinylidene fluoride membrane (03010040001). Then, the membrane was blocked by the Pierce™ Clear Milk Blocking Buffer (37587; Thermo Scientific™, USA) for immunological testing. After the removal of the blocking solution, the membrane was incubated with the primary antibody diluent at 37°C for 4 h, followed by the cultivation of the secondary antibody diluent at room temperature for 1.5 h. Later, the antibody diluent on the membrane surface was removed, and the membrane was covered with BeyoECL Plus luminescent solution (P0018S; Beyotime). After 1 min, the excess liquid was absorbed by the absorbent paper. Finally, the membrane was visualized and analyzed by the BIO-RAD Gel Doc XR gel imager. All the antibodies were purchased from Abcam or Sigma–Aldrich, where the primary antibodies mainly included those against UHRF1 (ab194236; 90 kDa; 1/1,000), COX26 (ab59020; 26 kDa; 1/1,000), Bcl-2 (ab182858; 26 kDa; 1/2,000), Bax (ab32503; 21 kDa; 1/4,000), DNMT1 (ab188453, 183 kDa, 1/1,000), DNMT3A (ab188470, 125 kDa, 1/1,000), DNMT3B (SAB2108295, Sigma, 86 kDa, 1/1,000), and glyceraldehyde-3-phosphate dehydrogenase (GAPDH) (ab8245; 36 kDa; 1/5,000), and the secondary antibodies were horseradish peroxidase-conjugated goat anti-rabbit IgG (ab6721; 1/6,000) and goat anti-mouse IgG (ab6728; 1/5,000).

### 2.9 MSP

MSP is a simple, specific, and sensitive way to detect single gene methylation. First, the DNA was extracted from the cochlear tissue with GenElute Mammalian Genomic DNA (G1N70; Sigma–Aldrich). In light of the operating instructions in the research of Ogunlaja et al. [22], Zymo EZ DNA Methylation-Lightning kit (D5030) was used to complete the sulfite treatment of DNA samples. In order to detect unmethylated (U) and methylated (M) alleles, COX26 MSP-specific primers were designed (Table 2). According to the requirements of the TIANGEN MSP kit (EM101), the amplification was conducted in the specific detection system. Ultimately, the reaction products were determined by agarose gel electrophoresis.

**Table 2:** Primers for MSP

**Gene**	**Forward primer (5**′**-3**′)	**Reverse primer (5**′**-3**′)
Connexin26-1	GTCCCTGTGGTGGACCTA	CCAGGCGTTACACTTCACCA
Connexin26-2	AGTTTGCAACAATGGACAAAAGC	GCCCAGGTTTCCCATAACCA
Connexin26-3	GGTCCCTGTGGTGGACCTA	ACCAGACGCTGCATGAAGAA
18Sribosomal RNA (rRNA)	CAGCAGCCGCGGTAATTCC	CCCGTGTTGAGTCAAATTAAGC

### 2.10 Chromatin immunoprecipitation (ChIP)

The cochlea containing Corti’s organ was treated with formaldehyde and glycine in a conventional manner to terminate the cross-linking reaction [23]. Then, the lysate was fully processed by ultrasound. The DNA concentration and fragment size were determined. The following immunoprecipitation process referred to the instructions of the ChIP Assay kit (P2078; Beyotime). In a nutshell, the collected samples were diluted at a ratio of 1/10. About 50 µL of the samples were stored as the control group and contained only magnetic beads. By contrast, the samples in other groups were cultivated with the UHRF1 polyclonal antibody (Catlog#PA5-29884; Invitrogen, USA) at 4°C for 1 h, followed by the incubation with the protein A/G magnetic beads at 4°C overnight. Subsequently, the DNA in the samples was extracted using the DNA extraction kit as the steps mentioned in the MSP method. The binding relationship between UHRF1 and COX26 was detected by PCR [23].

### 2.11 Cell counting kit (CCK)-8

HEI-OC1 cells were collected, washed after the treatment, and plated on a 96-well plate at a density of 10,000 cells/well. The CCK-8 reagent (40203ES76; YEASEN, China; 10 μL), which can undergo a reduction reaction with succinate dehydrogenase in the mitochondria of living cells, was added to HEI-OC1 cells for 4 h of incubation (conventional culture conditions). The 96-well plate was placed in a microplate reader to analyze the absorbance at 450 nm.

### 2.12 Flow cytometry

The HEI-OC1 cells after the external intervention were washed with pre-cooled PBS. 3 × 10^5^ HEI-OC1 cells were resuspended in 1× binding buffer (100 μL). Then, HEI-OC1 suspension was added with Annexin V-FITC (5 μL) to bind to phosphatidylserine, followed by being mixed with the nucleic acid dye propidium iodide in a dark environment. After 15 min, 400 μL 1× binding buffer was blended into the mixed suspension again. The mixture was added to a CytoFLEX flow cytometer (Beckman Coulter Life Sciences, USA) for detecting the HEI-OC1 apoptotic rate. The reagents mentioned in the above steps were all from the YEASEN Annexin V-FITC/PI Apoptosis Detection kit (40302ES60).

### 2.13 Statistical analysis

The analyses of experimental data were realized by GraphPad 8.0 software. In Figure 2, the difference between the data in both groups was compared by an independent sample *t*-test. The difference in the remaining results (≥3 groups) was analyzed by one-way analysis of variance and the Bonferroni post-test. Finally, *p* < 0.05 was considered to be statistically significant.

## 3 Results

### 3.1 IH increased the methylation of COX26 and the expression of UHRF1 in the cochlea of neonatal rats

**Figure 1:**
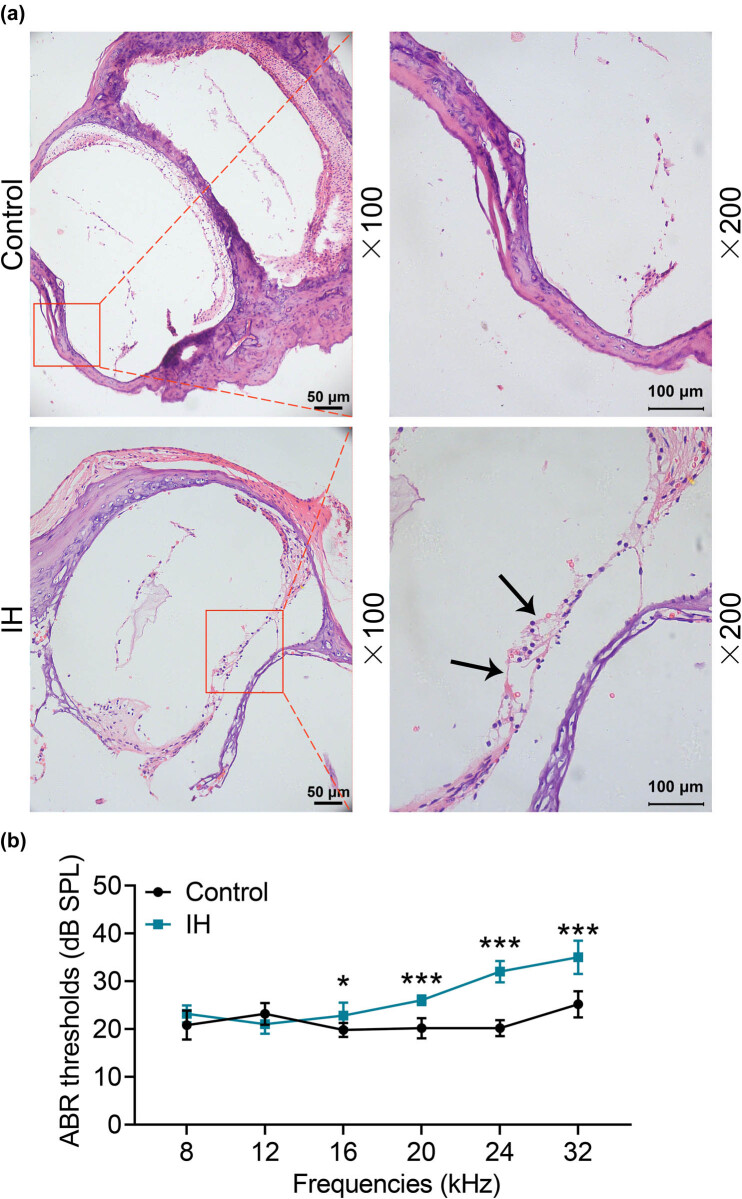
IH caused structural damage to the cochlea of neonatal rats. (a) HE staining was used to identify the pathological changes of the cochlea induced by IH. Magnification: ×100 and ×200. (b) The representative figure for the result of ABR threshold amplitudes in rats at six frequencies after exposure to IH.

From the pathological staining results in Figure 1a, we found that there were complete structures around Corti’s organ in the control group, such as spiral ligaments and cochlear basement membrane, and the outer hair cells were coherent. However, the Corti’s organ structure in the IH group was destroyed with the uneven distribution of hair cells. Furthermore, the thresholds of ABR in the IH group were significantly higher at 16–32 frequencies as compared with those in the control group (Figure 1b, *p* < 0.05). These findings indicated that IH treatment obviously damaged the cochlear structure.

**Figure 2:**
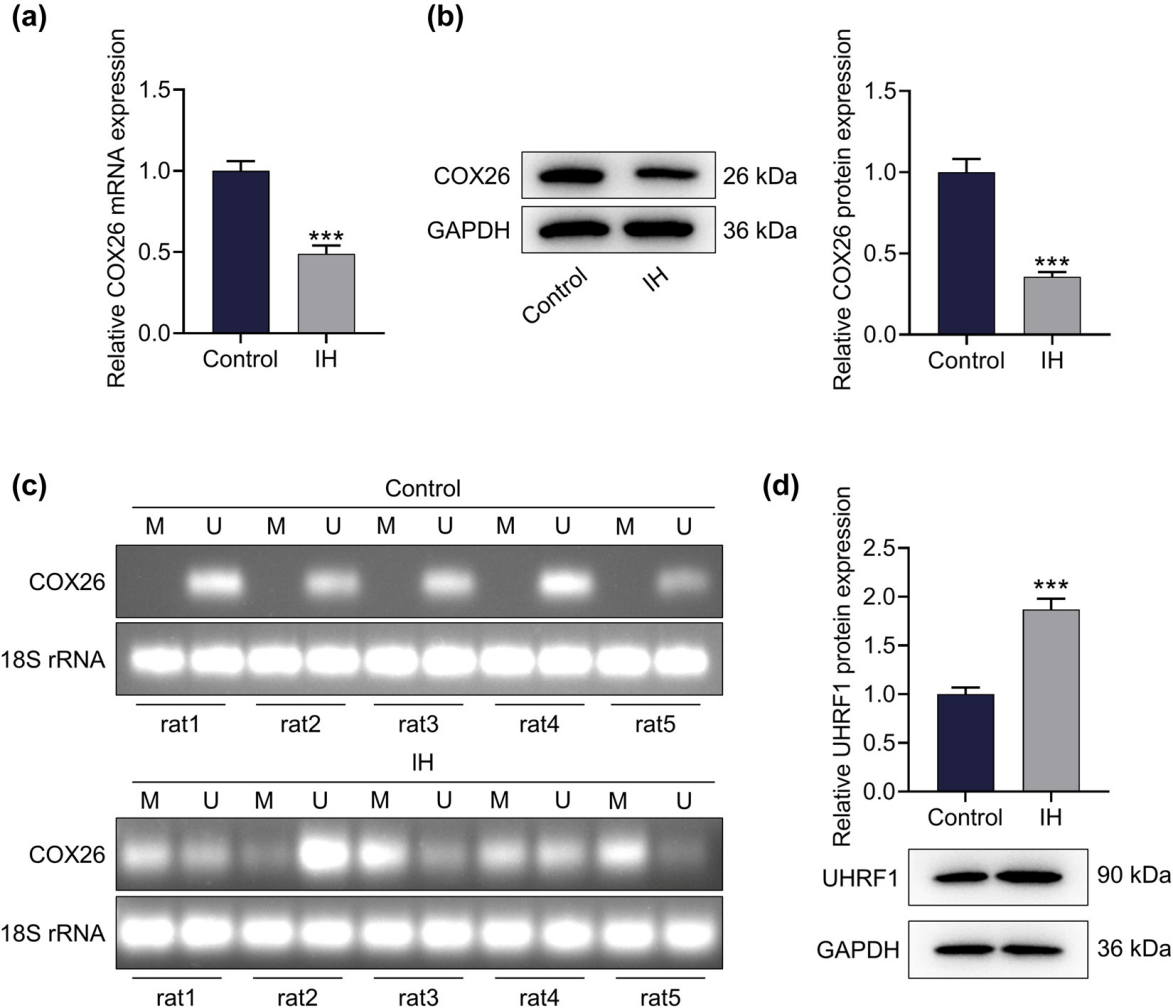
IH increased the methylation of COX26 and the expression of UHRF1 in the cochlea of neonatal rats. (a and b) The effect of IH on the expression of COX26 in the cochlea was tested by qRT-PCR (a) and Western blot (b). GAPDH was applied as an internal reference gene. (c) The effect of IH on the COX26 methylation level in cochlea tissue was analyzed by MSP. 18S rRNA was used as an internal reference gene. (d) The effect of IH on the expression of UHRF1 in the cochlea was detected by Western blot. GAPDH served as an internal reference gene. All experiments were repeated three times to average. ^***^ *p* < 0.001 vs control. Abbreviations: M, methylated; U, unmethylated.

Notably, COX26 expression was overtly lower in IH-injured cochlea tissues than in normal tissues (Figure 2a and b, *p* < 0.001). Specific PCR set for methylation levels showed that the methylation process of COX26 was markedly activated after IH induction (Figure 2c). At the same time, UHRF1, which regulated DNA methylation, was notably highly expressed under IH stimulation (Figure 2d, *p* < 0.001). Besides, except for DNMT3A, IH stimulation increased the expressions of DNMT3B and DNMT1, which catalyzed DNA methylation (Figure S1, *p* < 0.001).

### 3.2 CoCl_2_ caused damage to the Corti’s organ in the cochlea and reduced COX26 expression by promoting COX26 methylation

**Figure 3:**
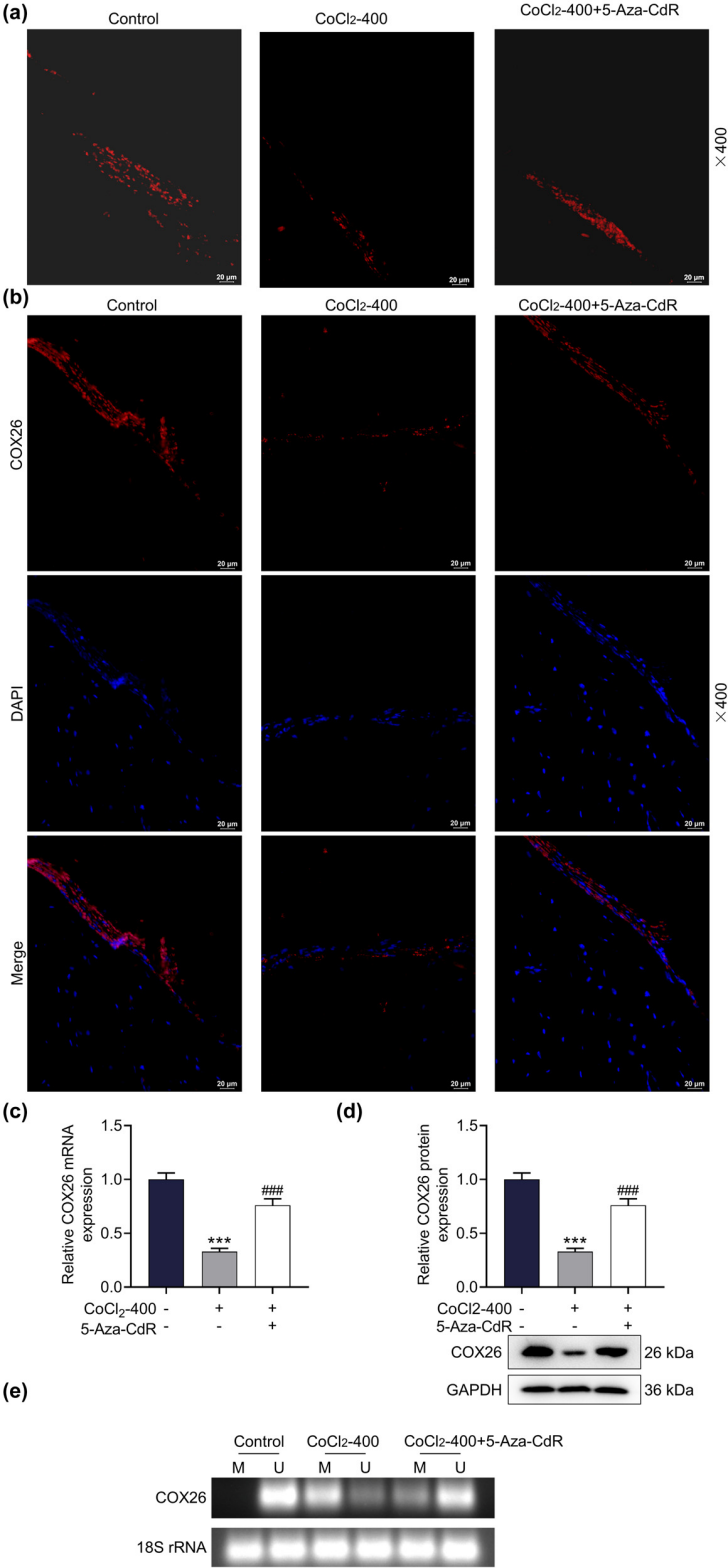
CoCl_2_ caused damage to the cochlea containing Corti’s organ and reduced COX26 expression by promoting COX26 methylation. (a) The effects of CoCl_2_ and 5-Aza-CdR on the structural integrity of the cochlea Corti’s organ and the loss of hair cells were observed by Phalloidin staining. Magnification: ×400. (b) The effects of CoCl_2_ and 5-Aza-CdR on the expression of COX26 in the Corti’s organ of the cochlea were observed by immunofluorescence staining. Magnification: 400×. (c and d) The effects of CoCl_2_ and 5-Aza-CdR on the expression of COX26 in the cochlea were quantified by qRT-PCR (c) and Western blot (d). GAPDH was utilized as an internal reference gene. (e) The effects of CoCl_2_ and 5-Aza-CdR on the COX26 methylation level in the cochlea were analyzed by MSP. 18S rRNA was employed as an internal reference gene. All experiments were repeated three times to average. ^***^ *p* < 0.001 vs control; ^###^ *p* < 0.001 vs CoCl_2_-400. Abbreviations: 5-Aza-CdR, methyltransferase inhibitor; CoCl_2_-400, 400 μM CoCl_2_ reagent; COX26, Connexin26.

We carried out *in vitro* experiments to observe the isolated cochlea and hair cells. In this part of the experiment, CoCl_2_ was used as a hypoxia inducer to treat the cochlea containing Corti’s organ, and the methyltransferase inhibitor 5-Aza-CdR was used to suppress the methylation process. From the Phalloidin staining results, it could be clearly seen that the continuity of the outer hair cells in Corti’s organ exposed to CoCl_2_ was disrupted, the intercellular space was apparently enlarged, and a large number of hair cells were lost (Figure 3a). The intervention of 5-Aza-CdR alleviated this damage and restored the number of hair cells (Figure 3a). In order to improve the accuracy of the results, a series of assays were conducted to detect the expression level of COX26. The results showed a high degree of consistency: CoCl_2_-induced hypoxia obviously downregulated the expres sion of COX26 in cochlear hair cells (Figure 3b–d, *p* < 0.001), which was partially reversed by 5-Aza-CdR (Figure 3b–d, *p* < 0.001). The reason for this situation was answered in the next MSP experiment. Collectively, CoCl_2_ activated the methylation of COX26, while 5-Aza-CdR prevented the methylation process in the explant (Figure 3e).

### 3.3 UHRF1 in cochlear hair cells could bind to COX26 and its expression was upregulated by CoCl_2_

**Figure 4:**
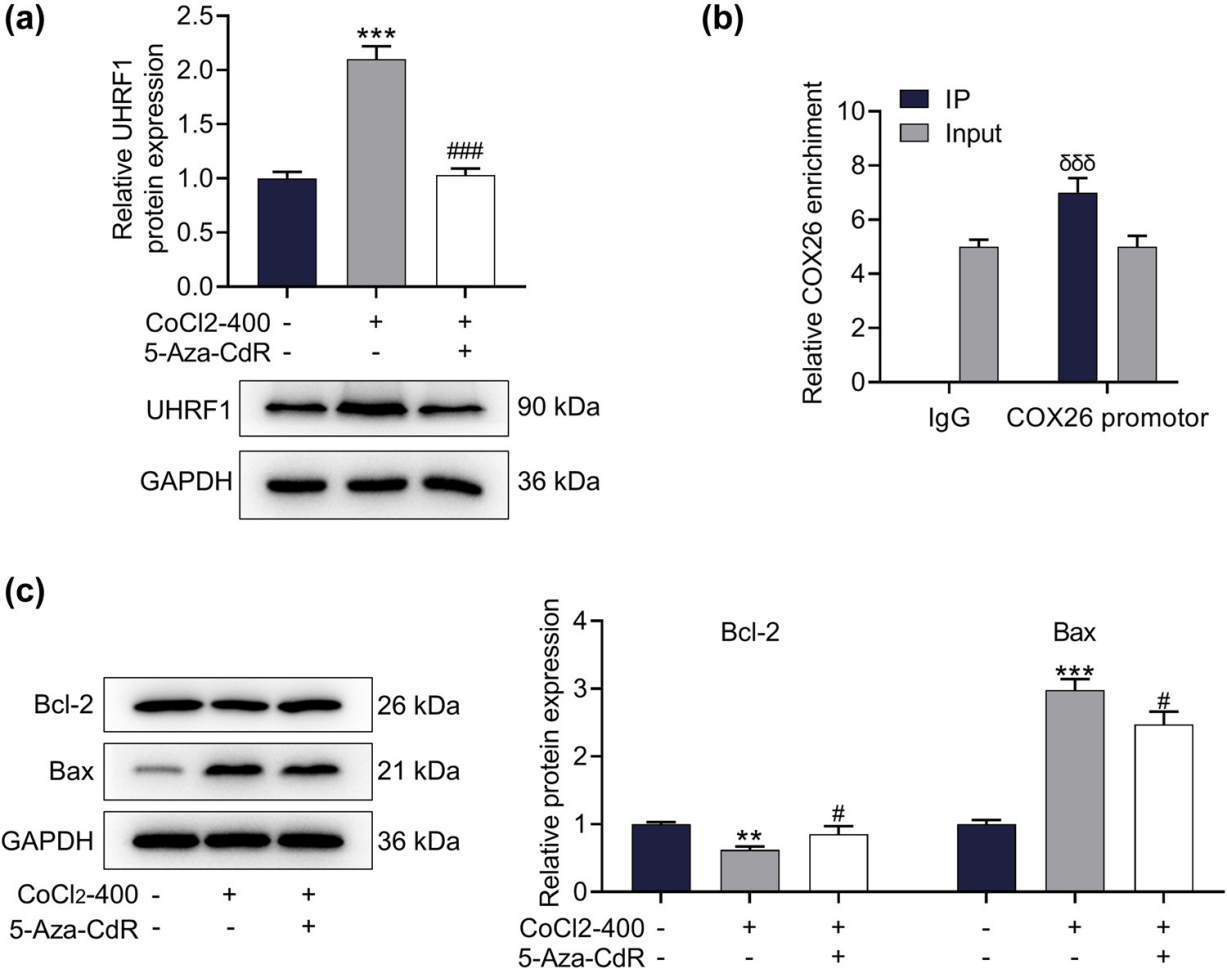
UHRF1 in cochlear hair cells could bind to COX26, and its expression was upregulated by CoCl_2_. (a) The effects of CoCl_2_ and 5-Aza-CdR on the expression of UHRF1 in the cochlea were detected by Western blot. GAPDH acted as an internal reference gene. (b) The binding of UHRF1 to COX26 in cochlear hair cells was analyzed by ChIP. (c) The effects of CoCl_2_ and 5-Aza-CdR on the expressions of apoptosis-related proteins in the cochlea were detected by Western blot. GAPDH was applied as an internal reference gene. All experiments were repeated three times to average. ^**^ *p* < 0.01, ^***^ *p* < 0.001 vs Control; ^σσσ^ *p* < 0.001 vs input^; #^ *p* < 0.05, ^###^ *p* < 0.001 vs CoCl_2_-400. Abbreviations: 5-Aza-CdR, methyltransferase inhibitor; CoCl_2_-400, 400 μM CoCl_2_ reagent; COX26, Connexin26.

In cochlear hair cells, the expression of UHRF1 was remarkably upregulated by CoCl_2_, which was reversed by the intervention of 5-Aza-CdR (Figure 4a, *p* < 0.001). The results of ChIP confirmed our conjecture that UHRF1 could bind to COX26 (Figure 4b, *p* < 0.001). Besides, the expressions of apoptosis-related proteins were also determined based on the loss of hair cells. It turned out that CoCl_2_ treatment could prominently upregulate Bcl-2 levels in cochlear hair cells and downregulate Bax expression. Moreover, 5-Aza-CdR treatment could effectively mitigate the changes of apoptosis-related proteins induced by CoCl_2_ treatment in cells (Figure 4c, *p* < 0.05).

### 3.4 UHRF1 knockdown inhibited COX26 methylation and promoted its expression in HEI-OC1 cells

**Figure 5:**
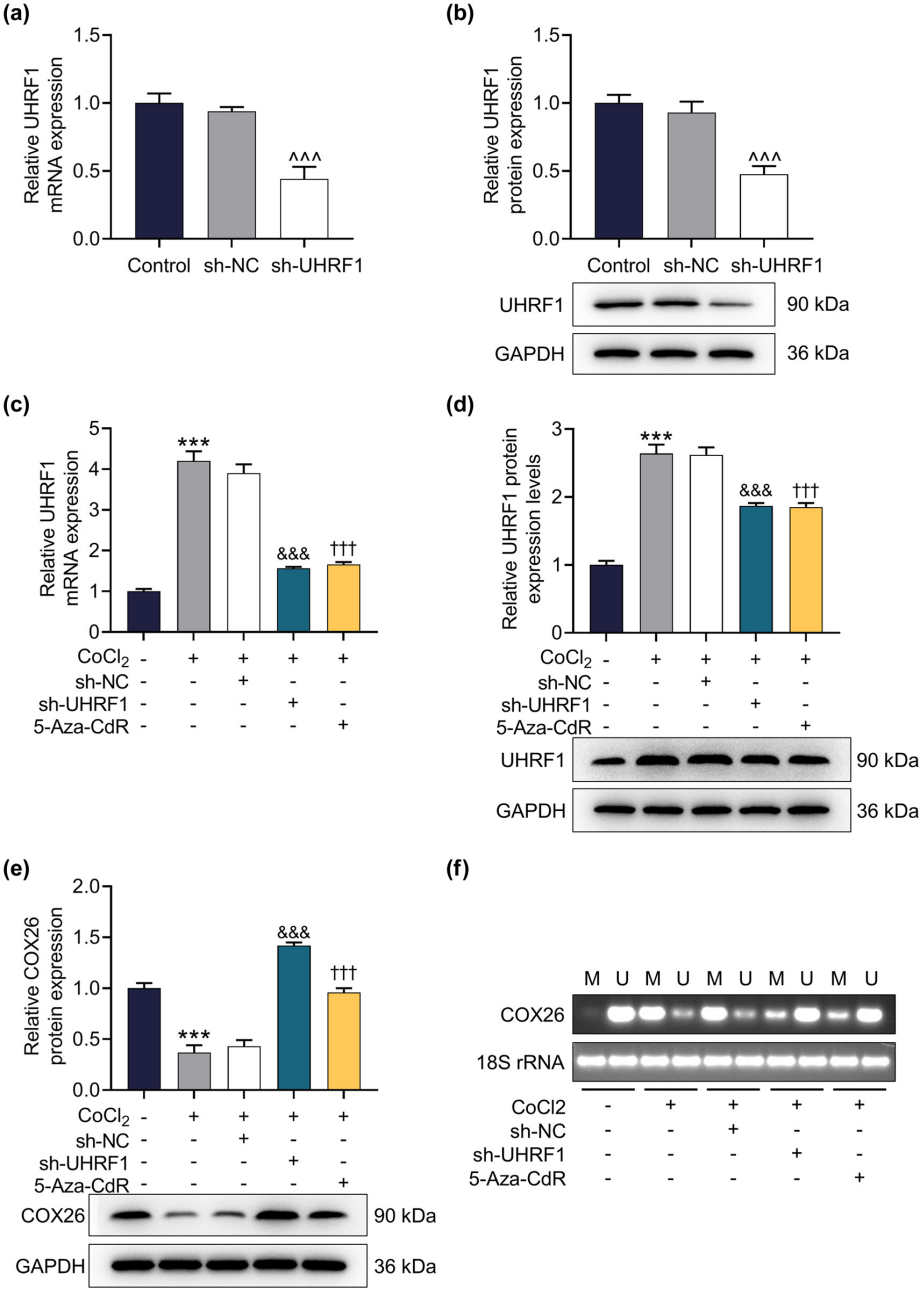
UHRF1 knockdown inhibited COX26 methylation and promoted its expression in HEI-OC1 cells. (a and b) The transfection efficiency of sh-UHRF1 was tested by qRT-PCR (a) and Western blot (b). GAPDH was used as an internal reference gene. (c and d) The effect of sh-UHRF1 on the expression of UHRF1 in HEI-OC1 cells induced by CoCl_2_ was tested by qRT-PCR (c) and Western blot (d). GAPDH was utilized as an internal reference gene. (e) The effect of sh-UHRF1 on COX26 expression in HEI-OC1 cells induced by CoCl_2_ was evaluated by Western blot. (f) The effect of UHRF1 knockdown on the COX26 methylation level in HEI-OC1 cells was analyzed by MSP. 18S rRNA was used as an internal reference gene. GAPDH was employed as an internal reference gene. All experiments were repeated three times to average. ^***^ *p* < 0.001 vs control; ^&&&^ *p* < 0.001 vs CoCl_2_ + sh-NC; ^†††^ *p* < 0.001 vs CoCl_2_. Abbreviations: 5-Aza-CdR, methyltransferase inhibitor; COX26, Connexin26.

In order to further understand the regulatory relationship between UHRF1 and COX26, an exogenous sh-UHRF1 plasmid was adopted. After transfection with sh-UHRF1, the mRNA and protein levels of UHRF1 in HEI-OC1 cells were conspicuously lessened (Figure 5a and b, *p* < 0.001). Moreover, CoCl_2_ treatment upregulated UHRF1 level but downregulated COX26 level, which was reversed by UHRF1 knockdown or 5-Aza-CdR treatment (Figure 5c–e, *p* < 0.001). Furthermore, we found that CoCl_2_ promoted COX26 methylation, which was offset by UHRF1 knockdown or 5-Aza-CdR treatment (Figure 5f).

### 3.5 Overexpressed COX26 partially alleviated the HEI-OC1 cell damage caused by CoCl_2_

**Figure 6:**
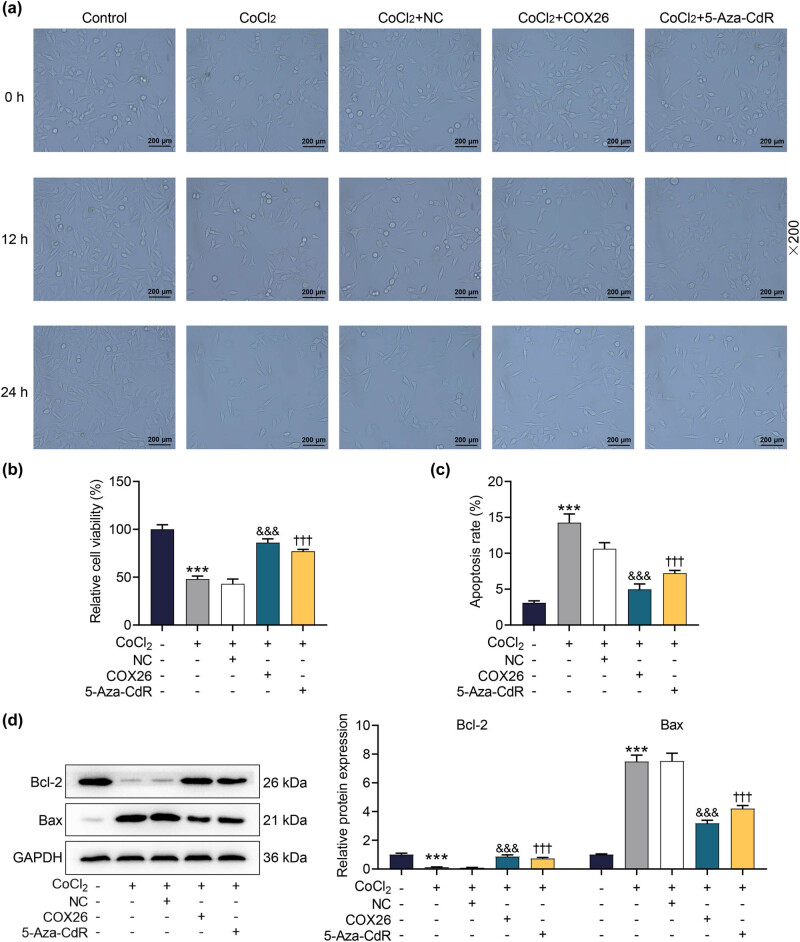
Overexpressed COX26 partially alleviated the damage of HEI-OC1 cells caused by CoCl_2_. (a) The effect of overexpressed COX26 on the fusion of HEI-OC1 cells induced by CoCl_2_ was examined under a microscope at a magnification of ×200. (b) The effect of overexpressed COX26 on the viability of HEI-OC1 cells induced by CoCl_2_ was assessed by CCK-8 assay. (c) The effect of overexpressed COX26 on CoCl_2_-induced HEI-OC1 cell apoptosis was tested by flow cytometry. (d) The effect of overexpressed COX26 on CoCl_2_-induced apoptosis-related protein expressions in HEI-OC1 cells was detected by Western blot. GAPDH was exploited as an internal reference gene. All experiments were repeated three times to average. ^***^ *p* < 0.001 vs Control; ^&&&^ *p* < 0.001 vs CoCl_2_ + sh-NC; ^†††^ *p* < 0.001 vs CoCl_2_. Abbreviations: 5-Aza-CdR, methyltransferase inhibitor.

**Figure 7:**
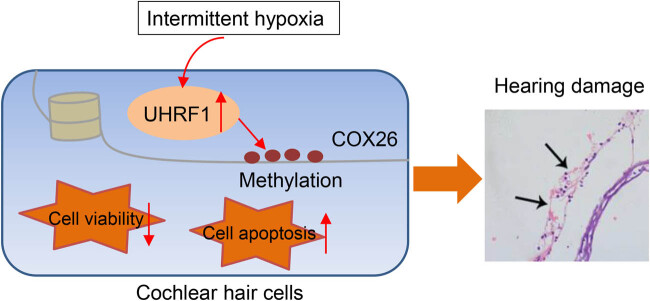
The schematic model of the mechanism for UHRF1 and COX26 interaction on the cochlear damage caused by IH.

Basic cell function experiments revealed that CoCl_2_-induced hypoxia reduced the fusion rate and viability of the cochlear hair cells while increasing the apoptosis of HEI-OC1 cells (Figure 6a–c, *p* < 0.001). Overexpressed COX26 could partially repair the aforementioned hypoxia-induced damage. To be specific, overexpressed COX26 downregulated the expressions of UHRF1 and Bax in CoCl_2_-induced HEI-OC1 cells but upregulated the levels of COX26 and Bcl-2 proteins (Figures 5c–e and 6d, *p* < 0.001). Meanwhile, overexpressed COX26 alleviated the basic functional damage of HEI-OC1 cells exposed to hypoxia (Figure 6a–c, *p* < 0.001). In addition, we found that the reversal effect of overexpressed COX26 was similar to that of 5-Aza-CdR (Figures 5c–e and 6a–d, *p* < 0.001). Figure 7 exhibited the schematic model of the mechanism for UHRF1 and COX26 interaction on the cochlear damage caused by IH. IH caused cochlear damage by suppressing COX26 expression through promoting UHRF1-mediated COX26 methylation, thereby leading to hearing damage (Figure 7).

## 4 Discussion

The human auditory nervous system consists of two parts, namely the peripheral auditory nervous system and the central auditory nervous system. Generally, the acoustic information in the external environment is transmitted to the cochlear structure of the inner ear via the peripheral auditory nervous system. Through the biological transformation of the hair cells on the cochlea Corti’s organ, mechanical signals are converted into neuroelectric signals and transmitted to the central nervous system [24,25]. It can be noted that Corti’s organ and the hair cells of the cochlea are the core structures of the peripheral auditory nervous system [25]. Cochlear tissue has unique physiological characteristics, such as a strong metabolism and a relatively simple blood supply of hair cells [26], which determines that the cochlear tissue is less tolerant to ischemia and hypoxia. In an ischemic hypoxic environment [19,27,28], blood flow is reduced due to cochlear vasospasm, which weakens the oxygen tension of the lymph located inside and outside the cochlea, resulting in damage to the helix of the entire hearing. The weakened activity of hearing-related enzymes causes reversible cell function damage and even irreversible damage in severe cases, causing damage to the hearing screw, cochlear hair cells, and the hearing system.

In the IH-induced model of this study, the structure of the cochlea was significantly damaged, substantial hair cells on the surface of Corti’s organ were lost, and the activity of hair cells was obviously inhibited; similar phenomena were also found in previous reports [17,19]. Conversely, a recent study by Park et al. [3] demonstrated that a high-fat diet or galactose injection treatment solely does not cause significant hearing loss; however, the intermediate effects are presented by the dual condition, and the maximum effects are presented by the triple-exposure condition after IH stimulation is conducted. This study also proved the important role of IH stimulation in the establishment of the hearing damage model in neonatal rats.

After a disease model was successfully constructed, we carried out a series of experimental analyses on the impact of UHRF1. UHRF1 is the “core protein” of epigenetic regulation, which can recognize the methylation regions of DNA and histones and combine with the corresponding enzymes to catalyze them. Besides, UHRF1 is the main factor influencing the stable inheritance of gene silencing in cell memory [29,30]. For example, Achour et al. found that UHRF1 and DNMT1 coexist in the molecular complex and could regulate the VEGF gene by downregulating p16^INK4A^ and RB1 levels, playing important roles in tumor angiogenesis and silencing tumor suppressor genes [31]. Babbio et al. also discovered that UHRF1 interacts with Suv39H1 and DNA methyltransferase, thereby silencing related tumor suppressor genes and ultimately promoting the occurrence and development of prostate cancer [32]. This study was the first to confirm the role of UHRF1 in hearing damage. When hearing damage occurred, UHRF1 in the cochlea/hair cells was abnormally highly expressed, causing a large loss of hair cells. Furthermore, ChIP and rescue experiments have indicated that the damaging effect of UHRF1 was related to the induction of COX26 methylation.

In mammals, COX26 is mainly distributed in the epithelium and connective tissues of the cochlea, such as supporting cells, intermediate cells, and basal cells [15]. COX26 has been identified as the most common and most widespread gene related to deafness [33]. There are also divergent opinions on the mechanism by which COX26 mutation causes hearing loss. In the earlier time, people speculated that hearing loss may attribute to COX26 gap junction dysfunction arising from cochlear endolymph K^+^ circulation disorder [34]. At the same time, some scholars believed that COX26 defective deafness may be elicited by cochlear developmental disorders [35]. Different from the past findings, our results confirmed that the inhibition of COX26 expression may be related to the abnormal hypermethylation induced by UHRF1.

Hearing impairment brings about immeasurable losses and a heavy burden to the development and growth of the child and the family [6]. The newborn hearing screening and follow-up genetic diagnosis work introduced in the 1990s have greatly reduced the incidence. The findings of this study may provide direct guidance on reducing the occurrence of genetic hearing impairment, helping to early screen out high-risk groups, diminishing the deafness disability rate, and decreasing the burden of deafness children, the family, and society in the long run. Furthermore, the findings may provide new ideas for the early and precise treatment of genetic hearing impairment and lay the foundation for the extensive development of drugs in the future.

## 5 Conclusion

This research carries out a range of experiments and finally determines that UHRF1 is highly expressed in IH-injured cochlea/hair cells and induces hypermethylation of COX26 when combined with COX26. Nevertheless, the current evidence is far from enough to support the clinical application of UHRF1 and COX26 in ameliorating hearing damage. Thus, further research is needed to clarify the molecular mechanism of UHRF1 aggravating hearing damage.

## Supplementary Material

Supplementary Figure
