# Supplementary material for: UHRF1-induced connexin26 methylation is involved in hearing damage triggered by intermittent hypoxia in neonatal rats
Source: Open Med (Wars). 2023 Feb 25;18(1):20230650. doi: 10.1515/med-2023-0650 (PMC9971736; doi:10.1515/med-2023-0650)
Supplement: Supplementary Figure [file med-2023-0650-sm.pdf]

Supplementary material

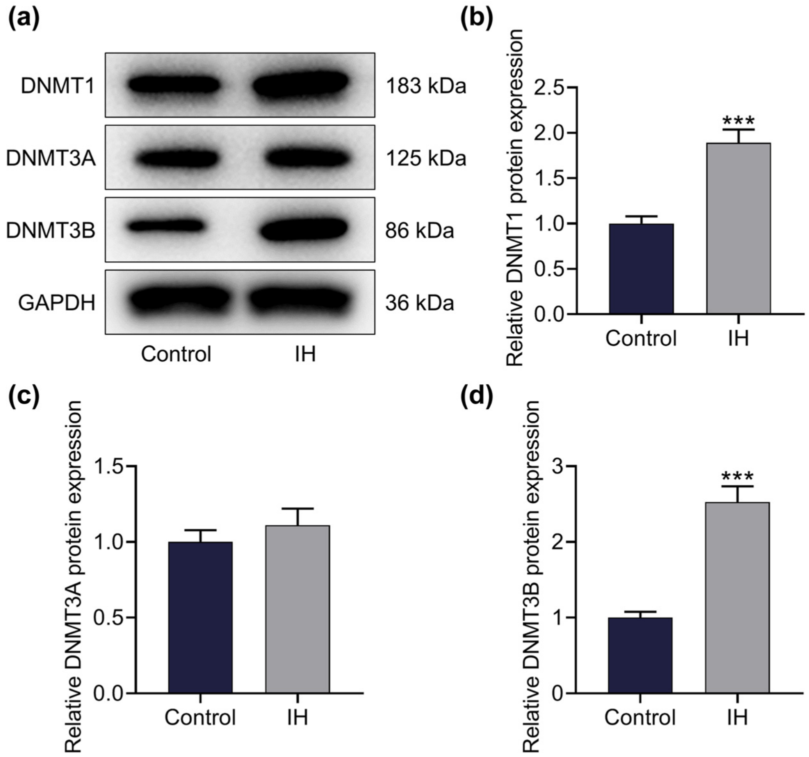

Figure S1: IH stimulation increased the expressions of Dnmt1 and Dnmt3b. (a) The effect of IH on the expressions of DNMT1, DNMT3A and DNMT3B in the cochlea was detected by Western blot. GAPDH was exploited as an internal reference gene. All experiments were repeated three times to average. Abbreviations: DNMT1, chemotactic DNA methyltransferase1; DNMT3A, DNA methyltransferases 3A; DNMT3B, DNA methyltransferases 3B; IH, intermittent hypoxia.
